# Validity and Reliability of the Caregiver Contribution to Self‐Care of Chronic Illness Inventory in Patients With Inflammatory Bowel Disease

**DOI:** 10.1002/jgh3.70340

**Published:** 2026-03-19

**Authors:** Silvia Cilluffo, Davide Bartoli, Daniele Napolitano, Valentina Biagioli, Antonello Cocchieri, Alessandro Monaci, Manuele Cesare, Piergiorgio Martella, Francesco Petrosino, Ercole Vellone

**Affiliations:** ^1^ Department of Biomedical Sciences for Health University of Milan Milan Italy; ^2^ Department of Wellbeing, Health and Environmental Sustainability Sapienza University of Rome Rome Italy; ^3^ CEMAD—Fondazione Policlinico Gemelli IRCCS Rome Italy; ^4^ Department of Biomedicine and Prevention Tor Vergata University Rome Italy; ^5^ Department of Medical and Surgical Sciences‐DIMEC University of Bologna Bologna Italy; ^6^ Section of Hygiene, Woman and Child Health and Public Health Gemelli IRCCS University Hospital Foundation Rome Italy; ^7^ Università Cattolica del Sacro Cuore Rome Italy; ^8^ Gemelli IRCCS University Hospital Foundation Rome Italy; ^9^ Direzione Professioni Sanitarie—ASL Salerno Salerno Italy; ^10^ Faculty of Nursing and Midwifery Wroclaw Medical University Wrocław Poland

**Keywords:** caregivers, chronic illness, Crohn's disease, inflammatory bowel disease, nurse education, nursing theory, psychometric properties, self‐care, ulcerative colitis

## Abstract

**Aim:**

To assess the validity and reliability of the Caregiver Contribution to Self‐Care of Chronic Illness Inventory (CC‐SC‐CII) among caregivers of patients with Inflammatory Bowel Disease (IBD).

**Design:**

A cross‐sectional psychometric study.

**Methods:**

This study is part of the multicenter longitudinal study project conducted in nine IBD centers. Factorial validity was evaluated through confirmatory factor analysis (CFA), and construct validity was examined through correlation with caregiver self‐efficacy. Internal consistency was assessed with omega coefficients and composite reliability. Test–retest reliability between baseline and 6 months was estimated using the Intraclass Correlation Coefficient (ICC).

**Results:**

A total of 275 informal caregivers of patients with IBD completed the CC‐SC‐CII. CFA supported the original three‐scale structure of the CC‐SC‐CII (maintenance, monitoring, management), with acceptable model fit after minor item‐level adjustments. Internal consistency ranged from 0.71 to 0.94 across subscales. ICCs ranged from 0.749 to 0.896, indicating good to excellent test–retest reliability. All item‐total correlations exceeded 0.30. Positive correlations with caregiver self‐efficacy scores supported construct validity.

**Conclusion:**

The CC‐SC‐CII is a valid and reliable instrument for assessing caregiver contribution to self‐care in the IBD population.

**Trial Registration:** ClinicalTrials.gov identifier: NCT06015789.

## 1 Introduction

Inflammatory bowel diseases (IBD), including ulcerative colitis (UC) and Crohn's disease (CD), are chronic immunological digestive conditions characterized by episodes of abdominal pain, persistent diarrhea, rectal bleeding, and weight loss [1].

Self‐care is a key component in the management of all chronic conditions, including IBD. In our recent work, we validated the Self‐Care of Chronic Illness Inventory (SC‐CII) for patients with IBD, demonstrating its reliability and validity in this specific population [2, 3, 4]. However, performing self‐care might be challenging for patients. In such cases, caregivers can play a valuable role in supporting patients with their self‐care efforts, known as caregivers' contribution to patients' self‐care (CCPSC).

CCPSC has been defined as “the provision of time, effort, and support on behalf of another person who is performing self‐care” [5]. Many studies conducted on CCPSC in chronic conditions have demonstrated that when caregivers contribute more to patients' self‐care, these patients have better outcomes in terms of quality of life and mortality [5, 6, 7, 8, 9, 10, 11]. However, to date, there have been no studies assessing the impact of CCPSC specifically on patients with IBD. This gap may be attributed to the absence of valid and reliable instruments available in the literature to measure this construct.

## 2 Background

The first instrument measuring CCPSC was the Caregiver Contribution to Self‐Care of Heart Failure Index (CC‐SCHFI).

Other instruments measuring the CCPSC were developed for ostomy care (the Caregiver Contribution to Self‐Care in Ostomy Patient Index) [12], diabetes (Caregiver Contribution—Self‐Care of Diabetes Inventory (CC‐SCODI)) [8], and COPD (Caregiver Contribution to Self‐Care of COPD Inventory) [10]. All these instruments have been inspired by the Middle‐Range Theory of Self‐Care of Chronic Illness, and they all measure the three dimensions of self‐care maintenance, self‐care monitoring, and self‐care management, as defined above [2, 4]. Moreover, all these instruments were developed, as for the CC‐SCHFI, by adapting the patient versions of the original instruments.

To measure CCPSC across various chronic conditions, the Caregiver Contribution to Self‐Care of Chronic Illness Inventory (CC‐SC‐CCI) was developed. This instrument is based on the Middle‐Range Theory of Self‐care of Chronic Illness and, with its 19 items, assesses caregiver contributions to self‐care in general chronic illnesses or in multiple chronic illnesses [13]. In similarity with the other caregiver contribution to self‐care instruments, the CC‐SC‐CII includes the three scales of Caregiver Contributions to Self‐Care Maintenance (7 items), Caregiver Contributions to Self‐Care Monitoring (5 items), and Caregiver Contributions to Self‐Care Management (7 items) [13].

So far, the CC‐SC‐CII has been tested only in informal caregivers caring for patients affected by at least one of various chronic illnesses, including heart failure, diabetes, stroke, and other long‐term conditions [14, 15, 16]. However, the instrument was never tested on caregivers of IBD patients. Such testing would be important because it would provide clinicians and researchers with an instrument to measure caregivers' contributions to IBD patients and to evaluate whether these contributions are associated with patients' and caregivers' outcomes.

## 3 Aim

This study aims to evaluate the validity and reliability of the CC‐SC‐CII in caregivers of IBD patients.

## 4 Methods

### 4.1 Design

For this paper, we used the baseline data from the ongoing longitudinal study “IBD‐SELF” [17]. The study was conducted in accordance with the Strengthening the Reporting of Observational Studies in Epidemiology (STROBE) guidelines [18] (Supporting Information).

### 4.2 Sample and Setting

In this study, we are enrolling IBD patients and their informal caregivers in nine IBD Units. Inclusion criteria for patients are: age ≥ 18 years, confirmed diagnosis of IBD and consent to participate. Patients are excluded from the study if they have received a diagnosis of IBD within the prior 12 months, have undergone IBD‐related surgery within the previous 6 months, have other severe chronic diseases or do not understand spoken and written Italian. Inclusion criteria per caregivers are: ≥ 18 years of age; recognized by the patient as the primary informal caregiver, either inside or outside the family network; providing the majority of informal care for the patient; willingness to participate in the study together with the respective patient; adequate spatial–temporal orientation; understanding of the Italian language.

### 4.3 Data Collection

In this study, data are collected on patients and caregivers during outpatient medical visits at baseline (T0), 6 months (T1), and 12 months (T2). Only baseline data from the first 250 enrolled caregivers were used for this study. Patients and caregivers are enrolled only after providing their approval and signing the consent form for participation. Participants complete the study battery (see below) at the end of the outpatient visit, which takes approximately 30 min for patients and caregivers. A research assistant is present during the questionnaire completion to provide support and clarify questions.

### 4.4 Instruments

The Caregiver Contribution to Self‐care of Chronic Illness Inventory (CC‐SC‐CII) [13] was used to measure informal CC to self‐care behaviors in patients with chronic illness. It is a 19‐item tool and contains three scales: the CC to self‐care maintenance scale, the CC to self‐care monitoring scale, and the CC to self‐care management scale. The CC to self‐care maintenance scale (7 items) asks the caregiver to report how often in the last month they recommended, assisted, or supported the patient in performing daily behaviors aimed at maintaining physical and mental stability. The CC to self‐care monitoring scale (5 items) assesses how often caregivers recommended that patients monitor signs and symptoms of chronic illness—i.e., checking for changes in bowel habits or the presence of abdominal pain. Finally, the CC to self‐care management scale (7 items) measures how likely caregivers are to help, assist, or recommend that patients respond to signs and symptoms of disease worsening, such as suggesting dietary modifications or contacting a healthcare provider when symptoms flare up. All responses for each scale's item use a 5‐point Likert format from 1 (never/not likely) to 5 (always/very likely), and all three scales have a score range between 0 and 100, with higher scores indicating better CC. A score of 70 or above is considered indicative of “adequate” self‐care.

The Caregiver Self‐Efficacy in Contributing to Patient Self‐Care (CSE‐CSC) scale [6, 9] is a valid and reliable instrument that assesses caregivers' confidence in their ability to support patients' self‐care. It consists of 10 items using a 5‐point Likert scale (1 = “not confident,” 5 = “very confident”). Also, this instrument has a 0–100 range score, with a higher score meaning greater self‐efficacy. This tool was used in the present study to test construct validity.

A sociodemographic and clinical questionnaire was used to gather information on participants' sociodemographic and clinical characteristics, including age, gender, education level, occupation, marital status, and employment status.

### 4.5 Statistical Analysis

Descriptive statistics (e.g., mean, standard deviation) were used to analyze participants' sociodemographic (Table 1) information and CC‐SC‐CII items (Table 2). CC‐SC‐CII items were also evaluated for skewness and kurtosis.

**TABLE 1:** Socio‐demographic characteristics of caregivers.

Variable	Total (*N* = 275)
Female	160 (58.2%)
Male	115 (41.8%)
Age (DS)	51.0 (13.20)
Education	
Primary School	7 (2.5%)
Middle School	61 (22.2%)
High School	134 (48.7%)
Bachelor's Degree	73 (26.5%)
Occupation	9 (3.3%)
Homemaker	29 (10.5%)
Retired	46 (16.7%)
Student	8 (2.9%)
Unemployed	8 (2.9%)
Active Worker	184 (66.9%)
Relationship with patient	
Husband or wife	161 (58.6%)
Parent	43 (15.6%)
Partner	12 (4.4%)
Friend	29 (10.5%)
Brother or sister	7 (2.5%)
None	23 (8.4%)

Abbreviation: IQR, interquartile range.

**TABLE 2:** Description statistics of the items composing the CC‐SC‐CII.

Caregiver contribution to self‐care maintenance						
How often do you recommend that the person you care for do the following things?						
Item	M	SD	Skewness	SE	Kurtosis	SE
Illness related behavior						
1. Make sure to get enough sleep?	2.70	1.29	0.03	0.15	−1.22	0.29
3. Do physical activity (e.g., take a brisk walk, use the stairs)?	3.08	1.24	−0.12	0.15	−0.81	0.29
7. Do something to relieve stress (e.g., mindfulness, yoga, music)?	2.87	1.48	0.14	0.15	−1.35	0.29
Health promoting behavior						
2. Try to avoid getting sick (e.g., flu shot, wash your hands)?	3.34	1.45	−0.38	0.15	−1.21	0.29
4. Eat special foods or avoid certain foods?	3.58	1.27	−0.67	0.15	−0.48	0.29
5. Keep appointments for routine or regular health care?	3.42	1.68	−0.43	0.15	−1.53	0.29
6. Take prescribed medicines without missing a dose?	3.39	1.65	−0.40	0.15	−1.50	0.29

Caregiver contribution to self‐care monitoring						
How often do you do the following things?						
Item	M	SD	Skewness	SE	Kurtosis	SE
8. Monitor the health condition?	3.56	1.44	−0.65	0.15	−0.89	0.29
9. Monitor for medication side‐effects?	3.58	1.41	−0.48	0.15	−1.13	0.29
10. Pay attention to changes in how one feels?	3.90	1.41	−1.03	0.15	−0.31	0.29
11. Monitor whether one tires more than usual doing normal activities?	3.64	1.38	−0.77	0.15	−0.60	0.29
12. Monitor for symptoms?	3.78	1.41	−0.89	0.15	−0.52	0.29

Contribution to symptom recognition						
	M	SD	Skewness	SE	Kurtosis	SE
13. If the person you care for has had symptoms in the past month, how quickly did you recognize them as symptoms of his/her illness?	—	—	—	—	—	—

Caregiver contribution to self‐care management						
When the person you care for has symptoms, how likely are you to recommend or perform symptom‐control behaviors?						
Item	M	SD	Skewness	SE	Kurtosis	SE
Autonomous behavior						
14. Change what the person you care for eats or drinks to make the symptom decrease or go away?	3.43	1.36	−0.30	0.15	−1.10	0.29
15. Recommend the person you care for to change the activity level (e.g., slow down, rest)?	3.23	1.27	−0.20	0.15	−0.87	0.29
16. Recommend the person you care for to take a medicine to make the symptom decrease or go away?	2.80	1.44	0.11	0.15	−1.32	0.29
17. Tell about the symptom to the healthcare provider of the person you care for at the next office visit?	3.73	1.36	−0.77	0.15	−0.59	0.29
Consulting behavior						
18. Call the healthcare provider of the person you care for to get guidance?	3.27	1.49	−0.26	0.15	−1.29	0.29
19. Think of a treatment you used the last time the person you care for had symptoms. Did the treatment you used make him/her feel better?	3.01	1.35	−0.77	0.15	0.08	0.29

*Note:* Item 13 serves as a bridge between self‐care monitoring and self‐care management.

Abbreviations: CC‐SC‐CII, caregiver contribution to self‐care of chronic illness inventory; M, mean; SD, standard deviation; SE, standard error.

CC‐SC‐CII factorial structure was tested using Confirmatory Factor Analysis (CFA). As performed in prior studies [19, 20], three separate CFAs, one for each scale, were performed. For the CC to self‐care maintenance scale, a two‐factor model: Health Promoting Behaviors with items #1, #3, #7, and Illness Related Behaviors with items #2, #4, #5, #6 was tested as reported in Table 2. For the CC to self‐care monitoring scale, a single‐factor model was tested, including items from #8 to #12 (Table 2). For CC to self‐care management, a two‐factor model was tested: Autonomous Behaviors with items #14, #15, #16, and #19; Consulting Behaviors with items #17 and #18 (Table 2). This factorial structure was the same as that which was tested in the original study [13]. Item #13 was removed from the CFA due to its assessment of symptom recognition. Given that symptom recognition does not align with either self‐care monitoring or self‐care management, descriptive analysis is more appropriate [21].

If strong correlations between factors were observed, a second‐order model was considered. Before conducting CFA, Mardia's test was used to check for multivariate skewness and kurtosis. As the data were not normally distributed, a robust estimator (i.e., robust maximum likelihood) was used in CFA. Model fit was assessed using several indices: comparative fit index (CFI) and Tucker‐Lewis index (TLI), with values ≥ 0.90 indicating good fit; standardized root mean square residual (SRMR) with values ≤ 0.08; and root mean square error of approximation (RMSEA) with values < 0.08 considered a good fit, together with the 90 Percent C.I. evaluation. A sample size of at least 200 patients was deemed sufficient for conducting an effective CFA [22].

Construct validity was examined by correlating scores from the three CC‐to‐self‐care scales (maintenance, monitoring, management) with the CSE‐CSC using Pearson's *r*. As per established guidelines, correlations of 0.10–0.29 were interpreted as weak, 0.30–0.49 as moderate, and ≥ 0.50 as strong. Reliability of the CC‐SCII was assessed using a multi‐method approach aligned with contemporary psychometric standards. For the multidimensional self‐care maintenance and management scales, composite reliability coefficients were calculated for each factor, and Omega Hierarchical (ωh) was used as an indicator of second‐order reliability. For the unidimensional self‐care monitoring scale, McDonald's Omega Total (ωt) was used in place of Cronbach's alpha to obtain more accurate estimates. Reliability values ≥ 0.70 were considered acceptable, and those ≥ 0.80 indicative of strong internal consistency. Item discrimination was evaluated using corrected item–total correlations, with coefficients ≥ 0.30 reflecting adequate discrimination.

To assess test–retest reliability, we utilized the Intraclass Correlation Coefficient (ICC) with a two‐way random‐effects agreement model. For this analysis, we correlated the scores of the CC‐SC‐CII scales collected at baseline with those collected at T1 (six months later). This approach provides valuable information about measurement stability beyond what internal consistency metrics alone can offer [23]. Following Koo and Li's (2016) guidelines, ICC values were interpreted as: poor reliability (< 0.50), moderate reliability (0.50–0.75), good reliability (0.75–0.90), and excellent reliability (> 0.90).

CFA and all statistical analyses for internal consistency measures were conducted using Mplus version 7.1 (Los Angeles, CA: Muthén & Muthén), while ICC analyses were performed using SPSS version 26.0 (IBM Corp., Armonk, NY, USA).

## 5 Results

### 5.1 Sample

The sample comprised 275 participants (58% female) with a mean age of 51 years (SD = 13.20). Most participants were employed (66.9%), 58.6% were married, and educational attainment was mainly at the high school level (48.7%), with 26.5% holding at least a bachelor's degree (Table 1).

### 5.2 Descriptive Statistics of the CC‐SC‐CII and Self‐Care Subscales

As shown in Table 2, several CC‐SC‐CII items exhibited non‐normal distributions (skewness/kurtosis > |1|), with the lowest mean scores observed for sleep promotion and medication recommendation, and the highest for symptom monitoring activities. Caregivers reported strong symptom recognition, with item 13 showing a high mean (M = 4.46) and 89.9% identifying symptoms quickly. As reported in Table 3, self‐care monitoring showed the highest mean (M = 67.57, SD = 31.91), followed by illness‐related and consulting behaviors. In contrast, health‐promoting and autonomous behaviors had the lowest mean scores, while self‐care maintenance and management showed intermediate levels.

**TABLE 3:** Scores of the SC‐CII and correlations with SCSES (*n* = 275).

	Mean	SD	SC‐SES (r)
Self‐care maintenance scale	55.39	25.34	0.07
Health promoting behavior	47.63	25.76	0.05
Illness related behavior	61.24	30.41	0.07
Self‐care monitoring scale	67.57	31.91	0.26**
Self‐care management scale	56.65	22.83	0.30**
Autonomous behavior	53.42	23.46	0.33**
Consulting behavior	62.80	32.73	0.15*

*Note:* SD, standard deviation; all scores are standardized 0–100; SC‐SES, self‐care self‐efficacy scale; correlations are significant at.

* *p* < 0.05.

** *p* < 0.01.

### 5.3 CC to Self‐Care Maintenance Scale

CFA testing of the CC to self‐care maintenance scale, comprising “health promoting behavior” (items #1, #3, and #7) and “illness related behavior” (items #2, #4, #5, and #6), did not initially yield supportive fit indices: *χ* ^2^ (13) = 46.091 (*p* < 0.001), CFI = 0.932, TLI = 0.890, RMSEA = 0.098 (90% CI = 0.068–0.129, *p* = 0.005), and SRMR = 0.054. Analysis of modification indices suggested adjusting the model by accounting for covariance between item #5 (Keep appointments for routine or regular health care) and item #6 (Take prescribed medicines without missing a dose). After this adjustment, model fit improved: *χ* ^2^ (12) = 38.210 (*p* < 0.001), CFI = 0.946, TLI = 0.905, RMSEA = 0.091 (90% CI = 0.059–0.124, *p* = 0.005), and SRMR = 0.043, indicating a good overall fit. All items showed significant factor loadings > 0.30.

**FIGURE 1:**
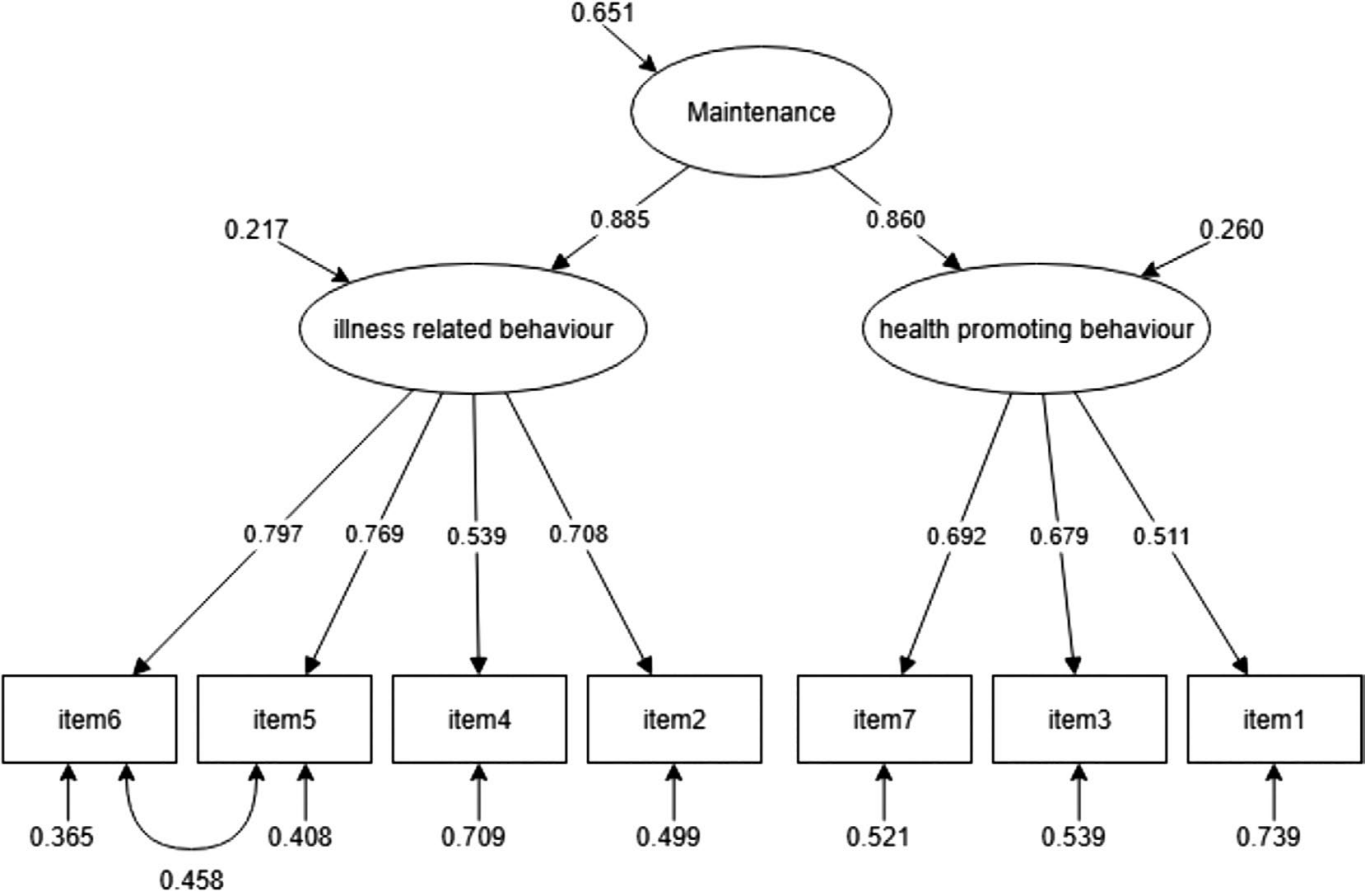
Confirmative factor analysis of Self‐Care Maintenance scale across CC‐SC‐CII. CC‐SC‐CII, caregiver contribution to self‐care of chronic illness inventory.

Given the high correlation between the two factors (*r* = 0.761), a second‐order factor representing the total self‐care maintenance score was specified. The model showed good fit (*χ* ^2^ (12) = 38.210, *p* = 0.0001; CFI = 0.946; TLI = 0.905; RMSEA = 0.091, 90% CI = 0.059–0.124, *p* = 0.019; SRMR = 0.043), with all factor loadings > 0.30 (Figure 1). Considering the multidimensional structure, ωh was 0.71. Composite reliability was 0.663 for “health promoting behavior” and 0.752 for “illness related behaviors,” with an overall multidimensional reliability of 0.880, indicating adequate reliability. Item–total corrected correlations were > 0.30, ranging from 0.426 (item #1) to 0.613 (item #2) for health‐promoting behavior and from 0.514 (item #4) to 0.729 (item #6) for illness‐related behavior. The ICC between CC to self‐care maintenance scores at the two timeframes was 0.804 (95% CI = 0.765–0.840, *p* < 0.001), confirming good test–retest reliability.

### 5.4 CC to Self‐Care Monitoring Scale

**FIGURE 2:**
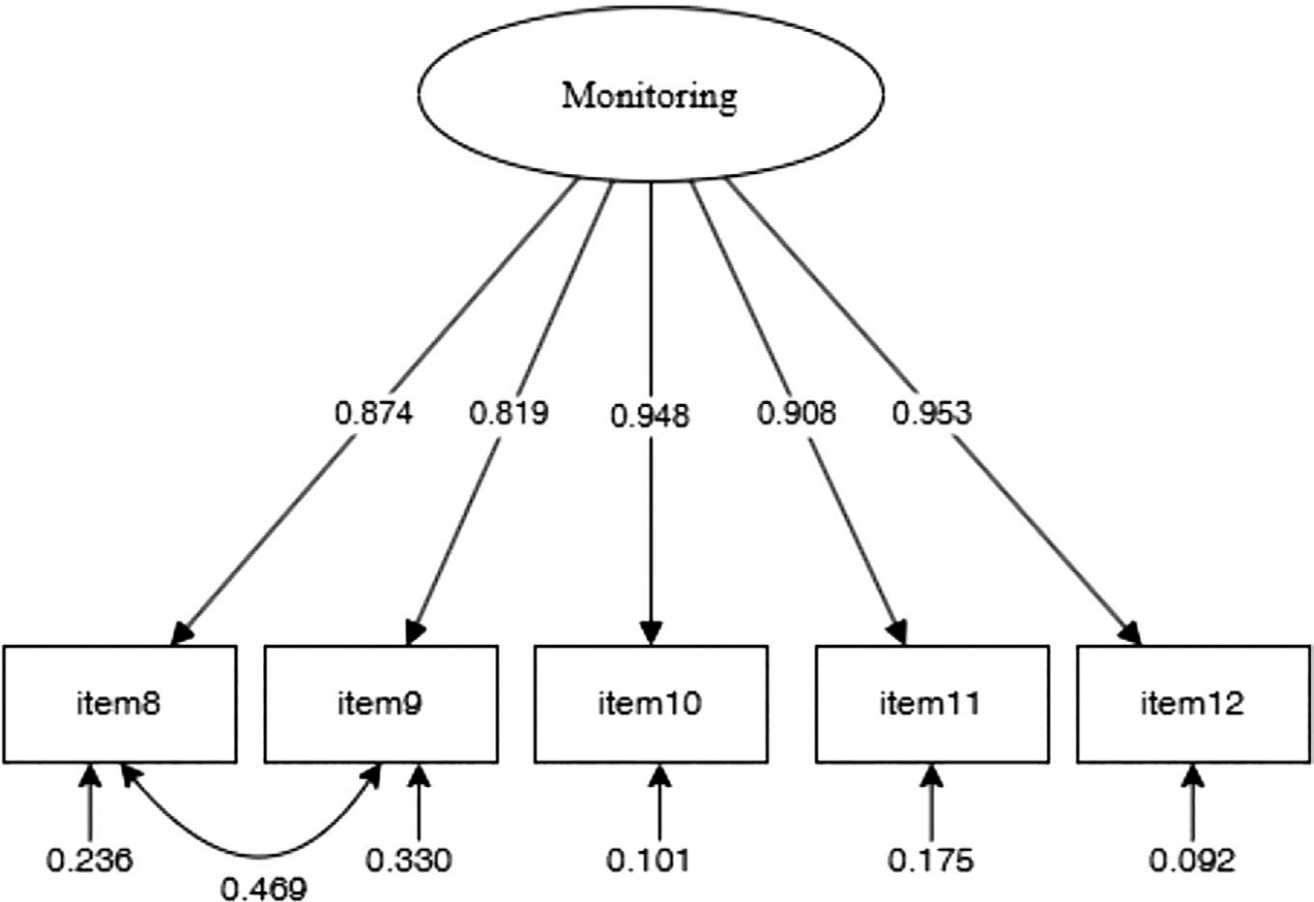
Confirmative factor analysis of Self‐Care Monitoring scale across CC‐SC‐CII. CC‐SC‐CII, caregiver contribution to self‐care of chronic illness inventory.

CFA of the CC to self‐care monitoring scale tested as a one‐factor model with item #8, #9, #10, #11, #12 in line with previous studies resulted with poor fit indices: *χ* ^2^ (5) = 37.905 (*p* < 0.001), CFI = 0.957 and TLI = 0.913, RMSEA = 0.157 (90% CI = 0.113–0.206, *p* = < 0.001), and SRMR = 0.025 [19]. After an inspection of the modification indices, a residual covariance between items #8 (Monitor the health condition) and item #9 (Monitor for medication side‐effects), consistent with a prior psychometric analysis [14], was allowed to correlate. The re‐specified model showed a better fit with the data, as evidenced by the following indices: *χ* ^2^ (4) = 8.583 (*p* = 0.072), CFI = 0.994 and TLI = 0.985, RMSEA = 0.066 (90% CI = 0.000–0.127, *p* = 0.273), and SRMR = 0.008. All items had significant factor loadings greater than 0.60 (Figure 2). Since the tested model was unidimensional, we computed the ωt, which was 0.94. The total correlation coefficients ranged from 0.824 (item #9) to 0.897 (item #10). The ICC value at the two timeframes considered was 0.896 (95% CI = 0.875–0.915, *p* < 0.001), confirming the good test–retest reliability of this scale.

### 5.5 CC to Self‐Care Management Scale

**FIGURE 3:**
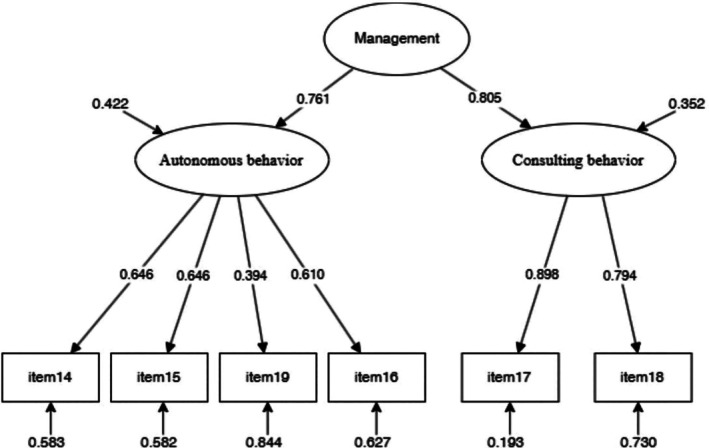
Confirmative factor analysis of Self‐Care Management scale across CC‐SC‐CII. CC‐SC‐CII, caregiver contribution to self‐care of chronic illness inventory.

CFA of the CC to self‐care management scale with the “autonomous behavior” (consisting of item #14, #15, #19, and #16) and “consulting behavior” (consisting of item #17 and #18) factors resulted in the following supportive fit indices: *χ* ^2^ (8) = 24.353 (*p* = 0.002), CFI = 0.951 and TLI = 0.908, RMSEA = 0.088 (90% CI = 0.049–0.129, *p* = 0.053), and SRMR = 0.039. Given the high correlation between the two factors (*r* = 0.610), we specified a second‐order factor to represent the total score of the self‐care management scale. The model showed a good fit with the data, as indicated by the following fit indices: *χ* ^2^ (8) = 24.348 (*p* = 0.002), CFI = 0.951 and TLI = 0.908, RMSEA = 0.088 (90% CI = 0.049–0.129, *p* = 0.053), and SRMR = 0.039. All items had significant factor loadings greater than 0.30 (Figure 3).

Given the multidimensional nature of the model under examination, ωh was calculated to be 0.88. The composite reliability for the “autonomous behavior” factor was 0.663, while it was 0.833 for the “consulting behavior” factor. The overall reliability of the multidimensional construct was determined to be 0.789, indicating adequate reliability for both factors. For the self‐care management scale, the item total corrected correlations ranged from 0.334 (item #19) to 0.600 (item #17), with all the values greater than 0.30. The ICC value was 0.749 (95% CI = 0.698–0.794, *p* < 0.001), confirming this scale's good test–retest reliability.

### 5.6 CC‐SC‐CII Scale

**FIGURE 4:**
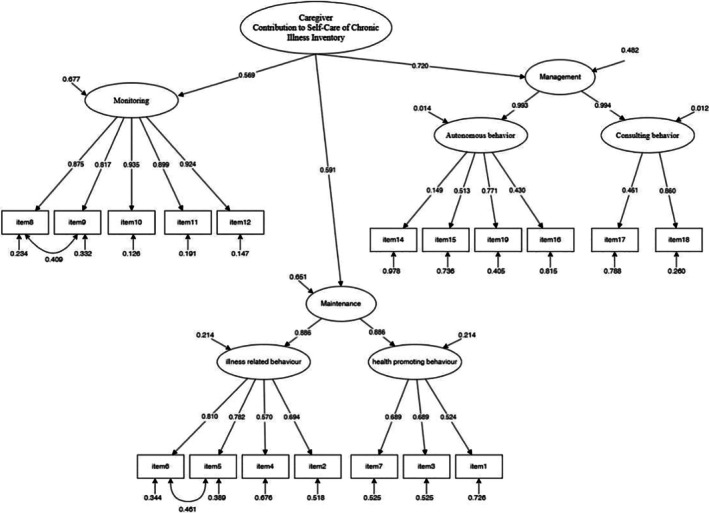
Confirmative factor analysis of CC‐SC‐CII. CC‐SC‐CII, caregiver contribution to self‐care of chronic illness inventory.

To demonstrate the emergent structure of the factors underlying the CC‐SC‐CII scales, a comprehensive CFA of the combined scales, dimensions, and respective items was conducted simultaneously. The analysis confirmed a comprehensive model, as indicated by the following indices: *χ* ^2^ (126) = 283.424 (*p* < 0.001), CFI = 0.932, TLI = 0.918, RMSEA = 0.067 (90% CI = 0.057–0.078, *p* = 0.004), and SRMR = 0.050. All items had significant factor loadings greater than 0.30 (Figure 4). Table 4 summarizes the global fit indices of the simultaneous CFA compared with prior validation of the CC‐SC‐CII. The fit indices observed in our IBD caregiver sample (CFI = 0.932, TLI = 0.918, RMSEA = 0.067, SRMR = 0.050) are highly comparable to those reported in the original version, supporting the cross‐cultural stability of the factor structure.

**TABLE 4:** Comparison of SC‐CII scores between the original validation and this study.

Version	N	*χ* ^2^ (df)	CFI	TLI	RMSEA (90% CI)	SRMR
Original	358	296.23 (142)	0.933	0.920	0.055 (0.046–0.064)	0.070
IBD (this study)	275	283.42 (126)	0.932	0.918	0.067 (0.057–0.078)	0.050

Construct validity was assessed by correlating the self‐care maintenance, self‐care monitoring, and self‐care management scores with those of the SC‐SES using Pearson's *r*. Self‐care monitoring showed a weak positive correlation with SC‐SES; self‐care management had a moderate correlation, but self‐care maintenance was not significantly correlated. Correlations between the SC‐SES and the dimensions of the SC‐CII ranged from weak to moderate. Specifically, weak correlations were found with self‐care monitoring (*r* = 0.26, *p* < 0.01) and consulting behavior (*r* = 0.15, *p* < 0.05). In contrast, moderate correlations emerged with self‐care management (*r* = 0.30, *p* < 0.01) and autonomous behavior (*r* = 0.33, *p* < 0.01). No significant correlations were observed for self‐care maintenance and its subdimensions (Table 3).

## 6 Discussion

This study evaluated the psychometric properties of the CC‐SC‐CII in caregivers of IBD patients, focusing on dimensionality, construct validity, and reliability. Findings indicate that the CC‐SC‐CII is both valid and reliable for use within the caregivers of IBD patients, consistent with results from studies on other chronic illnesses [14, 15, 24]. To our knowledge, this is the first study testing the psychometric properties of the CC‐SC‐CII within the IBD caregiver population, which is significant given the rising prevalence of IBD [25].

The highest‐scoring items were “Pay attention to changes in how one feels” (item #10), “Monitor for symptoms” (item #12), and “Tell about the symptom to the healthcare provider of the person you care for at the next office visit” (item #17), underscoring the central role that healthcare professionals, particularly nurses, play in the IBD care pathway [26]. Interestingly, while item #17 was among the lowest‐rated in the original CC‐SC‐CII validation study involving caregivers of patients with multiple chronic conditions [13], it ranked among the highest in our IBD‐specific sample, suggesting a greater reliance on healthcare providers for symptom management in this context.

The items with the lowest scores were “Make sure to get enough sleep”(item #1), “Do something to relieve stress (e.g., mindfulness, yoga, music)” (item #7), and “Recommend the person you care for to take a medicine to make the symptom decrease or go away” (item #16). This finding suggests that more efforts should be directed toward educating caregivers in IBD self‐management, since sleep quality and stress control are crucial for reducing symptoms and preventing flare‐ups [27, 28]. The low score on medication recommendation may reflect caregiver uncertainty in symptom management, highlighting the need for targeted education and support programs [29].

The CFA confirmed the original three‐scale structure, featuring two scales with two dimensions each (CC‐SC Maintenance and CC‐SC Management) and one scale with a single dimension (CC‐SC Monitoring), consistent with the foundational model [13] and cross‐cultural validations. On the Self‐Care Maintenance scale, our model revealed a high residual covariance between items #5 (routine healthcare visits) and #6 (prescribed medicines), similar to that observed in the Thai version [24]. This was different from the Chinese version [14], which reported the highest residuals between item #3 (physical activity) and item #4 (special diet). These findings suggest that, in our context, caregivers perceive healthcare visits and medication adherence as routine, closely interconnected behaviors. In the Self‐Care Monitoring scale, CFA of the one‐factor CC‐SC Monitoring scale confirmed a single‐factor structure in the self‐care monitoring domain [13], encompassing behaviors such as assessing symptoms and monitoring medication side effects.

Initially, the CFA produced a poor fit of the model, but after the adjustment with the correlation between the residuals of items #8 and #9, consistent with prior psychometric analysis [14], the fit of the model improved. The high correlation between the residuals of the two items might be explained by the lexical similarity within these two scale items.

The CC‐SC Management model fits well in the IBD caregiver context, displaying a second‐order structure with dimensions of CC to Autonomous Behavior and CC to Consulting Behavior, likewise the original model [13] and consistent with other psychometric analyses in other populations [24].

However, the IBD caregivers' model is satisfactory without requiring additional specifications as in the original model [13], whereas to achieve a good fit, they allowed the residuals of two items to correlate. These pairs included the caregiver's role in recognizing symptoms (item #12) and consequence of symptom management (item #19), changing diet or drinks (item #14), and adjusting activity level (item #15). The IBD CC‐SC Management model is represented by four items of Autonomous Behavior and two items of Consulting Behavior, differently from other psychometric analyses in different populations [24], but consistent with the SC‐CII original scale and theoretical framework [3, 30].

Our internal consistency results, tested with McDonald's Omega, were robust and comparable to those obtained in populations such as the Chinese and Thai versions [14, 24].

Finally, the study evaluated the construct validity of the CC‐SC‐CII by examining the correlations between self‐care and self‐efficacy. The findings revealed significant positive correlations across all self‐care dimensions and self‐efficacy, with the associations ranging from weak to moderate.

These results suggest the CC‐SC‐CII appropriately captures the construct of self‐care, aligning with previous research on chronic disease populations [20]. The correlation between self‐care and self‐efficacy was significant, though not consistent across all dimensions [31]. The findings further validate the construct validity of the SC‐CII for assessing self‐care levels in IBD caregivers. These results reinforce the generalizability of the CC‐SC‐CII's psychometric properties across diverse cultural and clinical contexts.

### 6.1 Strengths and Limitations

This study has several strengths: firstly, it was multicenter, including caregivers from different geographic locations across Italy. This diversity enhances the generalizability of our findings to Italian IBD. Furthermore, robust statistical methods provide a comprehensive insight into validity and reliability of the CC‐SC‐CII in this population.

However, this study also has some limitations: firstly, the use of a convenience sample may limit the generalizability of our findings, as it may only partially represent the broader population of IBD caregivers. Secondly, this was a cross‐sectional study design, so longitudinal studies are needed to establish whether improvements in caregiver contribution to self‐care are associated with better caregiver, patient, and dyadic outcomes. Temporal stability was assessed over a 6‐month retest interval, which is longer than the conventional 2–4 weeks. Thus, correlations indicate long‐term stability rather than classic test–retest reliability.

### 6.2 Recommendations for Further Research

Future research should explore the role of CC in self‐care on caregiver outcomes through longitudinal studies. Furthermore, it would be important to investigate which factors can predict a better level of CC to self‐care in IBD caregivers at the caregiver, patient, and dyad levels.

### 6.3 Implications for Policy and Practice

Since IBD prevalence is rising and these conditions mainly affect young‐mid‐age people [1], who usually have young‐mid‐age still employed caregivers, it is important to assess how caregivers can support patients' self‐care while dealing with these conditions together. For nurses and public health professionals, this instrument would help measure the caregiver's contribution to self‐care, allowing the tailoring of interventions to prevent caregiver burnout and strain. This instrument would allow IBD caregivers to self‐assess their efforts in contributing to self‐care and strengthen their ability to provide support while determining the care pathway [32, 33, 34, 35] and interventions to maintain patients' health. These findings validate the CC‐SC‐CII as a clinically valuable instrument for assessing and monitoring caregiver contributions to patient self‐care in IBD. Digital self‐management platforms could integrate the CC‐SC‐CII to provide real‐time caregiver feedback and facilitate healthcare provider identification of caregivers requiring additional support, thus enhancing the efficiency and personalization of caregiver‐oriented programs.

## 7 Conclusion

This study provides robust evidence supporting the validity and reliability of the CC‐SC‐CII in a new population: caregivers of IBD patients. The CFA confirmed the instrument's factorial validity, and its strong internal consistency and test–retest reliability over 6 months further reinforce its applicability in clinical and research contexts. The ability to measure caregiver contribution to self‐care offers a novel perspective in IBD care, where chronic disease management often involves complex, shared responsibilities. This validated tool can help clinicians identify caregiver needs and strengths, guide interventions, and promote caregiver‐inclusive care planning. Future research should investigate the predictive validity of this tool and its responsiveness to interventions aimed at enhancing caregiver engagement and dyadic self‐care.

## Disclosure

(a) The authors have checked to make sure that our submission conforms as applicable to the Journal's statistical guidelines described here. (b) The authors confirm that our submission conforms to the Journal's statistical guidelines. The author team includes statisticians, specifically Davide Bartoli (D.B.) and Francesco Petrosino (F.P.), who contributed their statistical expertise to this manuscript. (c) The author(s) affirm that the methods used in the data analyses are suitably applied to their data within their study design and context, and the statistical findings have been implemented and interpreted correctly. (d) The author(s) agree to take responsibility for ensuring that the choice of statistical approach is appropriate and is conducted and interpreted correctly as a condition to submit to the Journal.

## Ethics Statement

The study was conducted in accordance with Good Clinical Practice and the Revised Declaration of Helsinki. The Territorial Ethics Committee (Lazio 3) N. 0023486/23 of 02/08/2023 reviewed and approved the protocol. The study was also registered on ClinicalTrials.gov with the identifier NCT06015789.

## Consent

Before enrollment, all participants provided written informed consent. They also received comprehensive oral and written information about the study's aims and procedures.

## Conflicts of Interest

The authors declare no conflicts of interest.

## Supporting information


**Data S1:** Supporting Information.

## Data Availability

The data that support the findings of this study are available from the corresponding author upon reasonable request.
